# Antibacterial activity of methylated egg white proteins against pathogenic G^+^ and G^−^ bacteria matching antibiotics

**DOI:** 10.1186/s40064-016-2625-3

**Published:** 2016-07-04

**Authors:** Seham Abdel-Shafi, Ali Osman, Gamal Enan, Mona El-Nemer, Mahmoud Sitohy

**Affiliations:** Botany Department, Faculty of Science, Zagazig University, Zagazig, 44519 Egypt; Biochemistry Department, Faculty of Agriculture, Zagazig University, Zagazig, 44511 Egypt

**Keywords:** Egg white protein, Methylation, Antibacterial, Pathogenic bacteria, Antibiotic

## Abstract

Native egg white protein with high level of acidic amino acid residues (pI = 4.8) and hydrophilic nature was transformed into its methylated derivative (MEW), acquiring rather hydrophobic and basic character (pI = 8). The MIC of MEW against ten studied bacteria (G^+^ and G^−^) ranged between 0.5 and 1 μg/disc matching or excelling the comparative values of some known specific antibiotics (ranging from 1 to 7.5 μg/disc). Combinations of MEW (1 MIC) and different ready-made disc concentrations of antibiotics indicated either nil, antagonistic or synergistic antimicrobial effect. Replacing the antibiotic Ciprofloxacin by gradual levels of MEW (20–100 %) proportionally increased the potentiality to induce bigger sized inhibition zones. MEW (1 MIC) could inhibit the growth of 6 G^+^ and 4 G^−^ pathogenic bacteria in their liquid broth media during 24 h at 37 °C, indicating its broad and wide specificity. TEM examination indicated the susceptibility of the two types of bacteria (G^+^ and G^−^) to the antimicrobial action of MEW as manifested in different signs of cellular deformations, confirming its broad specificity and its mode of action was rather targeting the cell wall and cell membrane.

## Background

Antibiotics are gradually losing their antibacterial effectiveness over the last decades, as pathogens evolve resistance against them. The evolved resistant microorganisms are able to combat antibiotics, leading to ineffective treatment, persistence and spread of infections (Jyoti et al. 2014). Thus, antibacterial resistance continues to be a global public health concern, threatening the effectiveness of antibiotic therapy and posing challenges to the efforts of developing novel antibiotics. Morbidity and mortality issued from drug-resistant infections are on the rise all-over the world. WHO (The World Health Organization) has identified antimicrobial resistance as one of the three greatest threats to human health. WHO day in 2011 focused on “Combating Antibiotic Resistance” (WHO 2011).

Resistance was defined (Cloete 2003) as the temporary or permanent ability of an organism and its progeny to remain viable and/or multiply under conditions that would destroy or inhibit other members of the strain. Microorganisms are becoming extremely resistant to most existing antibiotics, especially Gram-negative rods (e.g., *Escherichia coli*, *Salmonella spp*, *Klebsiella oxytoca*, *Pseudomonas aeruginosa*, *Acinetobacter* spp), which are resistant to almost all currently available antibiotics. Resistance can be associated with virulence, acting as a potentially deadly duo, as observed in the recent large epidemic spreading of *E. coli* O104:H4 in Europe, notably in Germany (Buchholz et al. 2011), where the antibiotic pipeline has become extremely dry (Hughes 2011). Thus antibiotic treatment is currently contraindicated in *E. coli* infections.

Some natural products can meet the current challenge via presenting new antibiotic drug candidates escaping the developed antibiotic resistance. They are both a fundamental source of a new chemical diversity and an integral component of today’s pharmaceutical compendium. The demand for such natural antimicrobial compounds has greatly increased to counteract the rising bacterial resistance against the synthetic antimicrobial agents, particularly when they are highly active, non-toxic and least harmful to environment.

Cationic antimicrobial peptides are widespread throughout the animal and plant kingdoms and play a fundamental role in innate immune defense, both through direct antimicrobial activity and immunomodulatory effects (Lo and Lange 2015). Egg was reported to have antimicrobial activity arising partially from some egg yolk components such as immunoglobulin Y, but mostly from egg white components (Li-Chan et al. 1995; Kovacs-Nolan et al. 2005). Egg white contains various proteins that might be responsible for this antimicrobial activity, which has not yet been sufficiently assessed.

The main focus of the current research is to accentuate the antimicrobial effectiveness of the native egg white proteins through esterification, which imparts both cationic and hydrophobic character, the two major factors determining protein antimicrobial activity. The obtained products (native and modified) were quantitatively assessed for their antimicrobial actions against some pathogenic G^+^ and G^−^ bacteria as compared to known specific antibiotics. This work will participate in answering the questions if this modified product can partially replace some known antibiotics in new formulations without losing the antibiotic potentiality while adding some action against the antibiotic resistant strains.

## Methods

### Collection of pathogenic bacteria

Pathogenic Gram positive bacteria, i.e.; *Listeria monocytogenes* (LMG 10470), *Listeria ivanovi* (LMZ 11352), *Bacillus subtilis* (LMG 8029), *Bacillus cereus* (ATCC 14579), *Staphylococcus aureus* (DSM 1104) and *Streptococcus pyogenes* (EMCC 1774) and pathogenic Gram negative bacteria, i.e.; *Klebsiella oxytoca* (LMG 3055), *Pseudomonas aeruginosa* (LMG 8029), *Escherichia coli* (LMG 8223) *and Salmonella Typhi* (LMG 10395) were kindly obtained from the Laboratory of Microbiology, Department of microbiology, Faculty of Science and Faculty of Medicine, Zagazig University, Egypt. Stock cultures were frozen at −20 °C in glycerol. For experimental use, the bacterial cultures were maintained on nutrient agar slopes at 4 °C and sub-cultured every 4 weeks. The bacterial strains were cultivated on nutrient agar and brain heart infusion agar media (Monica 1985).

### Egg white preparation and analysis

Fresh hen eggs were obtained from the Farm of Agriculture Faculty, Zagazig University, Egypt. Eggs were hand broken and the whites were manually separated from the whole egg. The egg white was gently mixed with a magnetic stirrer for 30 min to reduce the viscosity and lyophilized. The resultant lyophilized protein was denoted NEW (native egg white). A sample of NEW was hydrolyzed with 6 N HCl at a ratio of 1/100 (w/v) for 24 h at 110 °C in a sealed tube. Amino acid composition was determined using HPLC system (Waters 510, USA), with PICO–TAG column. The determination was carried out at 38 °C, detection wavelength 254 nm and flow rate 1.0 ml per minute. Amino acid composition was reported as g per 100 g protein. The content of tryptophan was determined according to the method of Yust et al. (2004).

### Egg white esterification

The procedure of Sitohy et al. (2000) was used for esterifying NEW by dispersing a suitable amount (5 % w/v) in concentrated methyl alcohol (>99.5 %). The reaction mixture was kept at 4 °C under continuous stirring while adding an amount of hydrochloric acid equivalent to 50 molar ratio (mole acid/mole carboxyl group) at the reaction start. At the end of the reaction duration (10 h), the reaction mixture was centrifuged at 10,000*g* for 10 min at 4 °C and the resulting supernatant was discarded, replaced by an equal volume of methanol and well mixed before re-centrifuging under the same conditions. This washing step was repeated three times. The final precipitate was dissolved in an appropriate amount of distilled water and neutralized with appropriate aliquots of 1 N NaOH to pH 7.5. The obtained solution was dialyzed for 3 days against distilled water and then lyophilized. The lyophilized sample was denoted MEW (methylated egg white proteins) and kept at −20 °C until analysis. The esterification extent of proteins was quantified by the color reaction with hydroxylamine hydrochloride as developed by Halpin and Richardson (1985) and modified by Bertrand-Harb et al. (1991).

### SDS–PAGE

SDS–PAGE was performed on a discontinuous buffered system according to Laemmli (1970). The final composition of the separation gel was 0.375 mol Tris–HCl (pH 8.8) and 0.1 % SDS. Five milligrams of each protein (NEW or MEW) were dispersed in 1 mL of 0.03 M Tris buffer (pH 8.0) for 15 min with vortexing. The extract was then centrifuged for 10 min at 11.000 *g* and an aliquot of the supernatant (20 µL) was mixed with 20 µL of SDS-sample buffer, heated at 96 °C for 3 min. An aliquot (10 µL) of the final mixture was electrophoresed. After running at 10 mA on the stacking gel and 20 mA on the running gel, staining was performed with Coomassie Brilliant Blue R-250 dye (0.2 % solution, freshly prepared in 45 % methanol and 10 % glacial acetic acid and 45 % distilled water). Approximate molecular weights of the unknown bands were calculated by relating the distance migrated by these bands to the distances migrated by the bands of standard known molecular weights.

### Urea–PAGE

NEW and MEW were analyzed by Urea-PAGE in 3 and 10 % stacking and resolving gels, respectively, according to Williams and Evans (1980).

### Protein pH-solubility curves

Protein pH-solubility curves were assayed at the pH range of 2–10 according to the procedure outlined by Chobert et al. (1991). The iso-electric points were deduced from the protein pH-solubility curves as the pH at which the protein is the least soluble.

### Evaluation of antibacterial activity

The antibacterial activities of methylated egg proteins (MEW) and native egg proteins (NEW) were tested against Gram positive (*Listeria monocytogenes*, *Listeria ivanovii*, *Bacillus subtilis*, *Bacillus cereus*, *Staphylococcus aureus* and *Streptococcus pyogenes)* and Gram negative bacteria (*Klebsiella oxytoca*, *Pseudomonas aeruginosa*, *Escherichia coli* and *Salmonella Typhi*) using standard disc diffusion method and turbidity liquid media test (conventional broth dilution assay).

### Minimum inhibitory concentration (MIC)

Minimum inhibitory concentration (MIC) was determined using disc diffusion method or conventional broth dilution assay. Kirby–Bauer disk-diffusion method was applied by swabbing bacteria on the surface of brain heart infusion agar or nutrient agar plates. Then, paper discs of 6-mm in diameter, previously loaded with MEW at levels of 0.5, 1, 2, 4 and 10 μg/disc with appropriate distances separating them from each other. Likewise, other discs previously saturated with NEW at similar levels were laid on another plate. The plates were incubated at 37 °C for 24 h and the diameters of the inhibition zones (mm) were measured using a transparent ruler (Bauer et al. 1966; Ehinmidu 2003). The conventional broth dilution assay (Lana et al. 2006) against the previously mentioned G^+^ and G^−^ bacteria was carried out simultaneously.

MIC of an antimicrobial is taken as the lowest concentration (μg/disc) in case of the disc diffusion method or (μg/ml) in case of conventional broth dilution assay, that will inhibit the visible growth of a microorganism after overnight incubation. The MICs of some specific antibiotic were simultaneously assessed against the targeted bacteria as follows: Ciprofloxacin (*L. monocytogens*, *P. aeruginosa*, *S. typhimurium*), Chloramphenicol (*L. ivanovii*, *B. subtilis*). Penicillin G (*St. pyogenes*), Gentamycin (*K. oxytoca*, *E. coli*) and Vancomycin (*S. aureus*, *B. cereus*).

### Bacterial susceptibility to MEW: antibiotic mixtures

Five antibiotics (Ciprofloxacin, Chloramphenicol, Penicillin G, Vancomycin and Gentamycin) were selected for carrying out the antimicrobial susceptibility test by disc diffusion assay. Antibiotic-impregnated wafers and similar discs previously saturated with MEW solution at MIC concentration were used to determine the sensitivity of certain bacterium for each.

The bacteria were swabbed on the surface of brain heart infusion agar plates and antibiotic discs and MEW impregnated discs were placed on the top of the agar. Other discs were impregnated in MEW in addition to the previously loaded antibiotic and handled similarly. The plates were incubated at 37 °C for 24 h and the inhibition zones diameter (mm) were measured using a millimeter ruler (Bauer et al. 1966; Ehinmidu 2003) and the bacteria susceptibility was concluded according to the National Committee for Clinical Laboratory Standards (1999).

### MEW substituting ciprofloxacin against *L. monocytogenes*

Using standard disc diffusion method, Ciprofloaxacin (Cip) antibiotic was selected to determine its antibacterial activity against *L.monocytogenes*. Ciprofloxacin was substituted by MEW at varying percentages; i.e. 0.0, 20, 40, 60, 80 and 100 % of its starting concentration (100 μg mL^−1^) and the resulting substituted preparations were tested for their ability to produce inhibition zones against *L. monocytogenes.*

The bacteria were swabbed on the surface of six brain heart infusion agar plates. Paper discs, 6 mm in diameter, were loaded with amounts of the selected antibiotics (0.02, 0.05, 0.5, 1, 2, 2.5, 4, 5, 7.5, 10 μg/disc). The plates were incubated at 37 °C for 24 h and handled as previously mentioned.

### Bacterial growth curve (turbidity test)

Turbidity (A_600_) was used to estimate CFU/mL in brain heart infusion broth media suspensions as an indicator of the bacterial growth over time. MEW and NEW (at their MIC values) were added to the medium (10 ml) containing 100 μl G^+^ or G^−^ bacteria (10^9^ CFU/mL) and examined for their growth as compared to control (without adding any substance). All treatments were incubated at 37 °C for different time periods (0, 6, 12, 18 and 24 h) before measuring the turbidity.

### Transmission electron microscopy (TEM)

*Staphylococcus aureus* (G^+^) and *Pseudomonas aeruginosa* (G^−^) were selected as targets for MEW action and TEM analysis. The bacteria were grown on brain heart infusion broth and incubated at 37 °C to reach maximum level of 10^9^ CFU mL^−1^ which was subsequently diluted to 10^8^ CFU mL^−1^ using peptone buffer solution (0.1 % peptone plus 0.85 % NaCl).

MEW (25 μg mL^−1^) was added to bacterial cell suspensions except control and incubated at 37 °C for 4 h. After completing the incubation, the bacterial cells were treated for TEM analysis and images according to Sitohy et al. (2013). The number of intact cells and deformed cells were visually counted in ten fields and the averages were calculated. Based on the level of of intact cells, bacterial mortality rate was calculated using the following formula:

$${\text{Number}}\,{\text{of}}\,{\text{intact}}\,{\text{cells}}\,\left( {\text{control}} \right) - {\text{Number}}\,{\text{of}}\,{\text{intact}}\,{\text{cells}}\,\left( {\text{Treatment}} \right)\,\times\,100/{\text{Number}}\,{\text{of}}\,{\text{intact}}\,{\text{cells}}\,\left( {\text{control}} \right)$$

The rate of cellular deformation was calculated as follows:

$${\text{Number}}\,{\text{of}}\,{\text{deformed}}\,{\text{cells}}\,\left( {\text{treatment}} \right) \times 100/{\text{Number}}\,{\text{of}}\,{\text{intact}}\,{\text{cells}}\,({\text{treatment)}}$$

### Statistical analysis

All experiments were performed in triplicates and results were expressed by the mean plus the standard error. Data were statistically analyzed using ANOVA variance analysis through the general linear models (GLM) procedure of the statistical analysis system software (SAS version 9.1, SAS Institute, Inc., 2003). Least significant differences were used to separate means at *p* < 0.05.

## Results

### Protein characterization

**Fig. 1:**
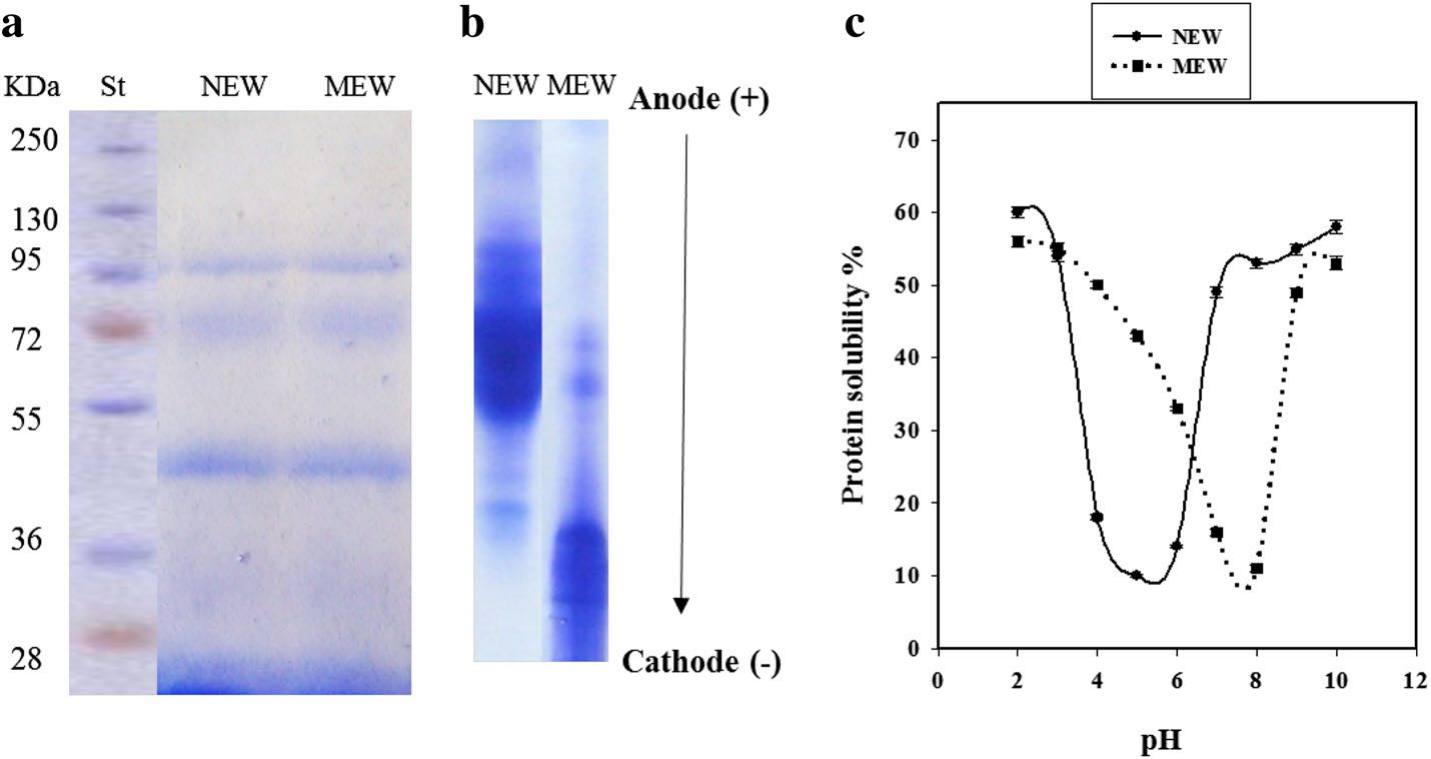
SDS-PAGE (**a**), Urea-PAGE (**b**) and pH solubility curves (**c**) at 2–10 pH range for native (NEW) and methylated egg white (MEW). St, Standard molecular weight markers

HPLC analysis indicated the presence of 18 amino acid in the composition of native egg white protein. The contents of the hydrophobic amino acid residues (Pro, Gly, Ala, Val, Ile, Leu, Phe) is around 41 % (3.3 + 3.9 + 6.6 + 7.1 + 5.5 + 8.5 + 6.1, respectively) of the total amino acid (data not shown). The content of the acidic amino acid residues (asp + glu; 10.3 + 12.5, respectively) is higher than that of the basic amino acids (arg + lys + his; 4.6 + 4.1 + 2.7, respectively), i.e.; 22.8 and 15.1 %, respectively (data not shown). The pH-solubility curve of the methylated egg white (MEW), indicated an alkaline isoelectric point around pH 8. Urea-PAGE indicated longer migration of MEW than NEW towards the cathode (Fig. 1).

SDS-PAGE of both NEW and MEW (Fig. 1) show only slight increases in the molecular weight of the modified protein. Both the native and modified forms (NEW and MEW) exhibited 4 bands corresponding to about 14, 45, 72 and 96 kDa, respectively. Generally, MEW did not show any toxicity on experimental Albino rats (data not shown). The LD50 of MEW was assumed to be more than 5000 mg/kg body weight/day which is regarded as practically non-toxic (data not shown).

### Magnitude of antibacterial action of MEW and NEW

The minimum inhibitory concentration (MIC) of MEW against the ten studied bacteria was 0.5 μg/disc aganist *L. monocytogenes* (G^+^) and *Klebsiella oxytoca* (G^−^) and 1 μg/disc aganist the other tested bacteria using agar well diffusion assay (Table 1). These values were equivalent to 10 and 25 μg/mL, respectively when using the conventional broth assay (data not shown). The native form of egg white (NEW) did not show any antibacterial activity at most used concentrations. The only exceptions were against *L. monocytogenes*, *L. ivanovii* and *B. cereus* where the MICs were relatively high, i.e., 4, 10 and 10 μg/disc, respectively (Table 1).

**Table 1:** The inhibition zones (mm) induced in Gram^+^ and Gram^−^ bacteria using disc assay under the influence of different levels (0–10 µg/disc) of native (NEW) and methylated (MEW) egg white and the associated MIC values (marked by *)

Microorganisms	Substance dose (µg/disc)									
	NEW					MEW				
	0.5	1	2	4	10	0.5	1	2	4	10
Inhibition zone (mm)										
Gram^+^										
*L. monocytogens*	–	–	–	12 ± 0.2*	14 ± 0.3	40 ± 0.9*	48 ± 1.2	50 ± 1.3	51 ± 1.4	53 ± 1.4
*L. ivanovii*	–	–	–	–	10 ± 0.1*	–	41 ± 1.0*	43 ± 1.0	47 ± 1.0	48 ± 1.2
*S. pyogenes*	–	–	–	–	–	–	42 ± 1.1*	45 ± 1.1	47 ± 1.1	50 ± 1.3
*S. aureus*	–	–	–	–	–	–	35 ± 0.7*	39 ± 0.8	43 ± 1.0	45 ± 1.1
*B. subtilis*	–	–	–	–	–	–	18 ± 0.3*	23 ± 0.5	23 ± 0.5	28 ± 0.7
*B. cereus*	–	–	–	–	5 ± 0.04*	–	12 ± 0.2*	12 ± 0.1	13 ± 0.3	16 ± 0.4
Gram^−^										
*K. oxytoca*	–	–	–	–	–	33 ± 0.6*	45 ± 1.1	48 ± 1.2	48 ± 1.1	50 ± 1.3
*P. aerugenosa*	–	–	–	–	–	–	18 ± 0.4*	20 ± 0.5	21 ± 0.4	25 ± 0.4
*S. typhimurium*	–	–	–	–	–	–	16 ± 0.3*	19 ± 0.3	20 ± 0.4	23 ± 0.4
*E. coli*	–	–	–	–	–	–	12 ± 0.2*	19 ± 0.4	20 ± 0.5	22 ± 0.3

For the sake of comparison, the MICs of some specific common antibiotics were assessed against the used bacteria, giving comparatively similar or higher MIC values. The MICs of Ciprofloxacin against three targeted bacteria (*L. monocytogens*, *P. aeruginosa*, *S. typhimurium*) were 2, 1 and 4 μg/disc, respectively. MICs of Chloramphenicol against *L. ivanovii*, *B. subtilis* were 7.5 and 2 μg/disc, respectively. MIC of Penicillin G against *St. pyogenes* was relatively very low, i.e. 0.02 μg/disc. MICs of Gentamycin against *K. oxytoca and E. coli* were 6 and 1 μg/disc, respectively and MICs of Vancomycin against *S. aureus and B. cereus* were 1 and 4 μg/disc, respectively (data not shown).

Comparing the tested substances to the reference antibiotics indicated that the MICs of MEW and NEW were 0.5 and 4 μg/disc against the G^+^ bacteria *L. moncytogenes*, respectively while Ciprofloxacin (Cip) was only 2 μg/disc (data not shown). Likewise, the MICs of MEW and NEW against the G^−^ bacteria *K. Oxytoca* were 0.5 μg/disc and unlimited value, respectively against 6 μg/disc for Gentamycin (data not shown). The MICs of MEW against the other microbes were 1 μg/disc (Table 1) which are still equivalent or lower than the specific antibiotics whose respective values ranged from 1 to 7.5 μg/disc (data not shown).

### Antibacterial activity of mixtures of MEW and antibiotics

In seven combinations of MEW (1 MIC) and different antibiotics (ANB), the mathematical sums of the inhibition zones induced by the individual components were always higher than the inhibition zone induced by their corresponding mixtures (Table 2). In one case (*Klebsiella oxytoca*), there was no difference between the sum of the inhibition zones of the individual components and the inhibition zone induced by their mixture. Only in two cases; i.e. G^−^ bacteria; *E. coli* and G^+^*B. cereus*, the sizes of the inhibition zones induced by the combination were higher than the sum of the inhibition zones induced by the individual components.

**Table 2:** Inhibition zones (mm) induced by of mixtures of antibiotic (ATB): MEW (methylated egg white) using disc diffusion assay

Microorganism	Antibiotic	Substance level (µg/disc)		Inhibition zone diameter (mm)		
		ATB*	MEW*	ATB	MEW	ATB + MEW
*L. monocytogenes*	Ciprofloxacin	2	0.5	15 ± 0.11	32 ± 0.55	37 ± 0.5
*Klesiella spp*	Gentamycin	6	0.5	15 ± 0.2	25 ± 0.38	40 ± 0.6
*S. pyogenes*	Pencillin G	0.02	1	29 ± 0.32	32 ± 0.47	36 ± 0.7
*L. ivanovii*	Chloramphenicol	7.5	1	19 ± 0.3	27 ± 0.33	31 ± 0.4
*S. aureus*	Vancomycin	1	1	14 ± 0.2	25 ± 0.4	32 ± 0.37
*B. subtilis*	Chloramphenicol	2	1	18 ± 0.3	7 ± 0.04	20 ± 0.43
*P. aeruginosa*	Ciprofloxacin	1	1	28 ± 0.45	7 ± 0.06	30 ± 0.5
*S. typhi*	Ciprofloxacin	4	1	18 ± 0.32	5 ± 0.03	20 ± 0.35
*E. coli*	Gentamycin	1	1	19 ± 0.3	5 ± 0.07	30 ± 0.44
*B. cereus*	Vancomycin	4	1	5 ± 0.1	4 ± 0.02	15 ± 0.22

* MIC against different microorganisms

In another experiment, the antibiotic Ciprofloxacin was substituted by varying extents of MEW (0.0, 20, 40, 60, 80 and 100 %) of its starting concentration (100 μg mL^−1^) and the resulting substituted preparations were tested for their ability to produce inhibition zones against *L. monocytogenes.* The results indicated respective inhibition zones equivalent of 32 ± 0.44, 33 ± 0.75, 34 ± 0.82, 35 ± 0.59 and 37 ± 0.85 mm. Replacing the antibiotic ciprofloxacin by equivalent gradual proportions of MEW induced bigger sizes of the inhibition zones, directly proportional to the ratio of MEW. The total substitution of the antibiotic with MEW induced relatively higher inhibition zone than the original antibiotic.

### Inhibition of the growth of pathogenic bacteria in liquid media

**Fig. 2:**
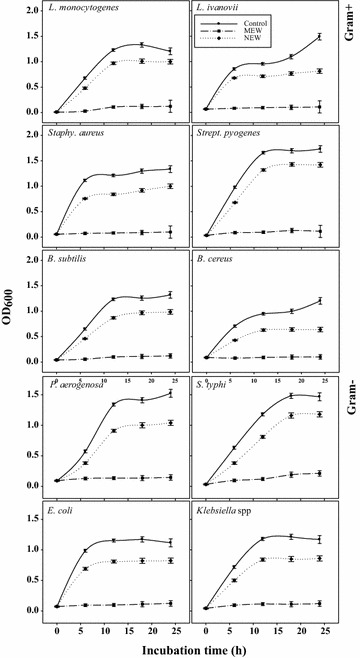
Growth curve of G^+^ and G^−^ pathogenic bacteria in brain heart infusion broth media during 24 h at 37 °C in the presence of 1 MIC of native (NEW) or Methylated egg white (MEW). Turbidity (A_600_) was taken as an indicator of the bacterial growth

Six pathogenic G^+^ bacteria were grown in their appropriate liquid media in the presence or absence of 1 MIC of MEW or NEW for 24 h at 37 °C (Fig. 2). The growth curves of the tested control bacteria reached a maximum turbidity after around 12 h at 37 °C. At this time point, MEW reduced the growth of G^+^ bacteria by about 90–94 %, while NEW inhibitory action was in the range 20–31 %. Similarly, MEW (1 MIC) could inhibit the bacterial growth of the four G^−^ by about 90–92 % against only 29–31 % reduction by NEW when grown at 37 °C (Fig. 2).

### TEM image analysis

**Fig. 3:**
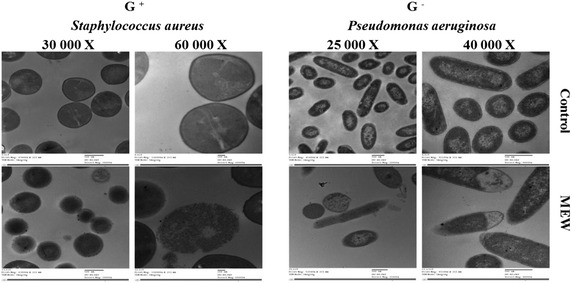
Transmission electron microscopy (TEM) of *Staphylococcus aureus* and *Pseudomonas aeruginosa* treated with 25 μg mL^-1^ of methylated egg white (MEW) at 37 °C for 4 h. Magnification powers were ×30,000 and ×60,000 for *Staphylococcus aureus* and ×25,000 and ×40,000 for *Pseudomonas aeruginosa*

*Staphylococcus aureus* (G^+^) and *Pseudomonas aeruginosa* (G^−^) (10^8^ CFU mL^−1^ in peptone buffer solution) were subjected to the action of 1 MIC (25 μg mL^−1^) of MEW for 4 h at 37 °C before subjecting to transmission electron microscope (TEM) analysis. The obtained TEM images (Fig. 3) of MEW-treated bacteria show various signs of cellular deformation indicating the direct disruptive action of MEW on the cell wall and cell membrane. The TEM images indicated that the presence of 1 MIC of MEW (25 μg mL^-1^) in the media of *Staphylococcus aureus* (G^+^) (OD_600_ = 0.5 at the time of application) has evidently reduced the relative content of the intact cells after 4 h of incubation at 37 ^◦^C producing a high mortality rate reaching 73 ± 4 %. Bacteria cells escaping the death were characterized by different manifestations of deformation. The ratio of the MEW-deformed bacteria relative to the total intact cells reached 60 ± 3 %.

MEW (1MIC = 25 μg mL^−1^) induced similar effects on *Pseudomonas aeurginosa* (G^−^) including high mortality rate (80 ± 5 %), high deformation rate (65 ± 3 %) and different signs of deformation. Deformed cells indicated cell shrinkage, cell membrane wrinkles and pore formation as well as some emptiness of cellular live material.

## Discussion

The amino acid composition of the native egg white proteins (NEW) with 41 % of hydrophobic amino acid residues (Pro, Gly, Ala, Val, Ile, Leu, Phe) indicates a hydrophilic nature. The relatively higher content of the acidic amino acid residues (glu + asp) over the basic amino acids (arg + lys + his) in NEW refers to an acidic nature, as confirmed by an acidic isoelectric point (ca. pH 4.8). The esterification of this protein resulted in the neutralization of the acidic amino acid residues, giving rise to a higher net positive charge as evidenced by higher isoelectric point (around pH 8) of the modified protein (MEW) and further confirmed by longer migration towards cathode on Urea-PAGE electropherogram as compared to NEW.

The conducted esterification might have resulted in the transformation of most of acidic amino acid residues (90 %) from the hydrophilic (carboxylate) into a hydrophobic form (methyl-carboxylate), i.e. more hydrophobic nature. SDS-PAGE showed only slight molecular weight increases in the modified proteins, due to the low molecular weight of the grafted methyl groups. The four bands revealed by the SDS-PAGE electrophergrams for NEW and MEW correspond to lysozyme, ovalbumin and ovotransferrin, respectively while the fourth band may indicate some aggregation between two smaller molecular weight subunits. Some of these components are originally active against bacteria in the native egg white (Valenti et al. 1982) and may thus participate in the antibacterial action of the modified form (MEW).

Hence, the major changes in the modified protein (MEW) are the increased positivity and hydrophobicity which can together promote the antibacterial activity of the modified protein. The majority of antimicrobial proteins are cationic and amphipathic with both hydrophilic and hydrophobic domains enabling their binding to the lipid components (hydrophobic region) and phospholipid groups (hydrophilic region) on the bacterial cell membranes (Jenssen et al. 2006).

The results of agar well diffusion assay indicated concentration dependent inhibition zone diameters for MEW. The minimum inhibitory concentration (MIC) of MEW against the ten studied bacteria was relatively very low (0.5–1 μg/disc) as compared to NEW (10 μg/disc). The MIC data indicated some closeness in antibacterial effectiveness between MEW and different antibiotics while NEW was mostly much less effective.

These relatively lower values of MIC of MEW against some microbes compared to some specific antibiotics (1–7.5 μg/disc) may indicate equivalent or higher antibacterial effectiveness. The only exception was with Penicillin G against *Streptococcus pyogenes* which manifested very low MIC (0.02 μg/disc) due to the particular susceptibility of this bacterium to Penicillin during the past 70 or 80 years (Horn et al. 1998; Ciftic et al. 2003).

Being protein in nature, the use of MEW as partial or total substitute to some antibiotics will not potentially incur any hazard in accordance with our initial toxicity data (unpublished). Comparing the effectiveness of this modified protein to other modified ones, e.g.; methylated soybean protein and methylated chickpea protein against *Listeria monocytogenes* and *Salmonella Enteritidis* (Sitohy et al. 2013) indicates its superiority.

The presence of synergistic effects between MEW and two antibiotics (Gentamycin and Vancomycin) may create the potentiality of formulating mixture of antibiotics and MEW opening the door for new therapeutic strategy with less use of the synthetic antibiotics. Replacing the antibiotic Ciprofloxacin by MEW at different percentages indicated that increasing the proportion of MEW increased proportionally the potentiality to induce bigger sized inhibition zones against the tested bacteria. This is evidently sound since the MIC of MEW (0.5 μg/disc) is lower than that of the antibiotic (2 μg/disc). This result may open the door to prepare partially or totally active protein-substituted antibiotic formulations. The ability of Modified proteins to act synergistically with antibiotics could be a new approach to solve the problem of resistant and less susceptible bacteria, particularly when MEW has broad-spectrum antimicrobial activity. This approach may have triple objective, i.e.; to aid the antibiotic escape the evolved resistance, to broaden the specificity of the antibiotic preparation and to attenuate the hazard of the synthetic drugs.

In accordance with the results of agar well diffusion, the growth curves of the tested control bacteria (using conventional broth dilution assay) at 37 °C after 12 h indicated that MEW reduced the growth G^+^ and G^−^ bacteria by about 90–94 and 90–92 % against only 20–31 and 29–31 % in case of NEW, respectively. These results agree with previous ones concluded for other cationic methylated proteins (Sitohy and Osman 2011; Sitohy et al. 2013).

TEM images indicated that MEW was associated with relatively reduced content of the intact cells, indicating higher mortality rates (73–80 %) in G^+^ and G^−^ bacteria (*Staphylococcus aureus* and *Pseudomonas aeruginosa*), respectively. The various signs of cellular deformation manifested of MEW-treated G^+^ and G^−^ bacteria (*Staphylococcus aureus* and *Pseudomonas aeruginosa*) indicate direct disruptive actions of MEW on the cell wall and cell membrane. This result goes in line with other different studies on methylated proteins (Friedrich et al. 2000; Sitohy et al. 2013). Cationic antimicrobial peptides were reported to target bacterial cell membranes and cause disintegration of the lipid bilayer structure (Shai 2002; Zhang et al. 2001). The potential attack of the cationic proteins on cell membranes and walls passes apparently through electrostatic interactions between the positively charged fragments of the cationic proteins and the negatively charged regions of cell wall or cell membrane emerging from teichoic acid component and phospholipid constituent, respectively (Murzyn et al. 2005) and a hydrophobic bonding between the alike hydrophobic regions of the antimicrobial MEW and cell membrane phospholipids.

## Conclusions

Chemical analysis of native egg while proteins (NEW) revealed higher level of acidic amino acid residues, acidic (pI = 4.8) and hydrophilic nature. Transformation of these protein into the methylated form did not significantly change their molecular weight (SDS-PAGE) but endowed them with rather hydrophobic and basic character (pI = 8 and longer migration on Urea-PAGE) which is potentially engendering antibacterial action.

The MIC of MEW ranged between 0.5 and 1 μg/disc (equivalent to 10–25 μg mL^−1^) against ten tested bacteria (G^+^ and G^−^) using agar well diffusion assay and the conventional broth dilution assay, respectively. The native form of egg white (NEW) did not show any antibacterial activity at most used concentrations. The only exceptions were against *L. monocytogenes*, *L. ivanovii* and *B. cereus*, where the MIC values were relatively high.

The ability of the modified proteins to act synergistically with antibiotics can be a new approach to solve the problem of bacterial resistance and less susceptible bacteria, particularly when MEW has a broad-spectrum antimicrobial activity, enabling it to partially replace the synthetic antibiotic to significant extents. This approach may have triple objective, i.e.; to aid the antibiotic escape the evolved resistance, to broaden the scope of the antibiotic action and to attenuate the hazard of the synthetic drugs.

The observed increase in the antibacterial activity of ciprofloxacin by gradual substitution levels of MEW may open the door to prepare partially or completely substituted active antibiotic preparations.

MEW has broad antibacterial specificity based on its inhibitory activity against all the studied pathogenic bacteria (6 G^+^ and 4 G^−^) and as confirmed by the TEM images indicating the susceptibility of the two types of bacteria (G^+^ and G^−^) to the antimicrobial action of MEW (methylated egg white proteins).
